# bcl-2 overexpression combined with p53 protein accumulation correlates with hormone-refractory prostate cancer.

**DOI:** 10.1038/bjc.1996.526

**Published:** 1996-10

**Authors:** I. Apakama, M. C. Robinson, N. M. Walter, R. G. Charlton, J. A. Royds, C. E. Fuller, D. E. Neal, F. C. Hamdy

**Affiliations:** University Urology Unit, Newcastle upon Tyne, UK.

## Abstract

**Images:**


British Journal of Cancer (1996) 74, 1258-1262
© 1996 Stockton Press All rights reserved 0007-0920/96 $12.00

### bcl-2 overexpression combined with p53 protein accumulation correlates with hormone-refractory prostate cancer

I Apakama[1], MC Robinson[2], NM Walter[2], RG Charlton[2], JA Royds[3], CE Fuller[3], DE Neal[1] and FC Hamdy[1]

[1]*University Urology Unit, Newcastle upon Tyne;* [2]*Department of Pathology, Freeman Hospital, Newcastle upon Tyne;* [3]*Department of Pathology, University of Sheffield, UK.*

**Summary** Seventy-seven men with histologically proven and newly diagnosed prostate cancer were investigated for the presence of bcl-2 protein overexpression and p53 protein accumulation by immunohistochemistry. Forty-five men had evidence of locally advanced and metastatic disease and were treated by means of hormone manipulation. Twenty-eight patients either failed to respond to initial hormone manipulation or relapsed within 37 months from diagnosis (median 20 months). Of the 77 cancers, 37 (48%) showed bcl-2 overexpression at diagnosis. Twenty-seven of those were treated with androgen ablation and 20 (74%) had hormone-refractory disease ($P=0.0128$). Twenty-three of 77 men (29.8%) had nuclear staining for p53 protein. Twenty-one of those were treated with hormone manipulation and 14 (66.6%) showed hormone resistance ($P=0.0012$). Seventeen patients had both bcl-2 overexpression and p53 protein accumulation, 16 of whom were hormonally treated, with 13 (81.2%) having hormone-refractory disease ($P<0.0001$). These findings suggest that the combined detection of p53 protein accumulation and bcl-2 overexpression may be useful in predicting hormone resistance in prostate cancer. By deregulating programmed cell death, alterations in these genes may prevent patients from responding to androgen ablation, or allow them to escape hormonal control of the disease.

**Keywords:** prostate cancer; apoptosis; p53; bcl-2; tumour-suppressor gene; oncogene

Adenocarcinoma of the prostate is the third most common malignancy in men in England and Wales, with over 8000 men dying from the disease every year (OPCS, 1993). Prostate cancer is unpredictable in its clinical course and biological behaviour. In Europe, over 50% of patients present with locally advanced and/or metastatic disease, amenable to palliation only. In these patients, androgen ablation remains the treatment of choice. However, approximately 15% of men will not respond to hormone manipulation, and the majority of those who do respond will relapse within 3 years. Hormone-refractory disease, while remaining unpredictable, is an untreatable condition resulting in considerable morbidity and mortality. Understanding the mechanisms by which a hormone-sensitive tumour escapes control, and altering these mechanisms to treat affected patients remains a challenge for clinicians and scientists alike.

The accumulation of alterations to both cellular oncogenes and tumour-suppressor genes (TSGs) is associated with tumorigenesis (Bishop, 1991; Marshall, 1991). The biological behaviour of a tumour and its response to treatment has been shown to be associated with alteration in expression of these oncogenes and TSGs.

In prostate cancer, it is now evident that hormone manipulation achieves its effect through activation of programmed cell death, otherwise known as apoptosis (Kyprianou *et al.*, 1990; Kerr, 1994; Colombel *et al.*, 1992). Apoptosis is a distinct mode of cell death, which occurs in tissues under normal physiological conditions and in disease, including cancer. A number of genes are responsible for the regulation of apoptosis in disease and in health, including the proto-oncogene bcl-2 (Hockenbury *et al.*, 1990; Bissonnette *et al.*, 1992; Korsmeyer, 1992) and the tumour-suppressor gene p53 (Lane, 1992; Shaw *et al.*, 1992). Bcl-2 is a protein which protects cells from going into apoptosis. The encoded protein is expressed in the cytoplasm of basal epithelial cells in normal and hyperplastic prostatic tissue. It is also normally expressed in lymphocytes. Hormone-refractory prostatic carcinomas characteristically possess high levels of bcl-2 protein expressed diffusely throughout the tumour. This is also seen in prostatic intraepithelial neoplasia (Colombel *et al.*, 1993). Bcl-2 overexpression appears to enable the prostate cancer cells to survive in an androgen-deprived environment, and to confer resistance to androgen withdrawal therapy. An untreated prostatic carcinoma with a high proportion of bcl-2-positive cells may, therefore, possess an inherent ability to become hormone refractory as these cells are protected from apoptosis following androgen ablation.

Nuclear accumulation of p53 protein has been shown to be strongly associated with p53 mutations. Wild-type p53 is involved in regulation of cellular proliferation by inducing apoptosis in response to DNA damage, whereas p53 in its mutant form is not able to mediate this effect. A significant proportion of primary human prostatic carcinoma show increased p53 nuclear protein accumulation, which appears to correlate with androgen independence (Navone *et al.*, 1993; Aprikian *et al.*, 1994).

Based on the evidence that bcl-2 and p53 serve, respectively, a repressor and effector function of a common cell death pathway, the aim of this study was to investigate the combination of bcl-2 protein overexpression and p53 nuclear protein accumulation, and their significance in relation to frequency of apoptotic bodies, tumour behaviour and clinical outcome following treatment of various stages of prostate cancer. The results and possible value of these two apoptosis-regulating factors in predicting hormone refractory disease are discussed.

### Patients and methods

### *Patients*

Seventy-seven men with histologically proven and untreated prostatic carcinoma were studied. Their age ranged from 46 to 88 years (median 71 years). Specimens were obtained from transurethral resection specimens as well as trucut biopsies before treatment. One sample was taken from a radical

Correspondence: FC Hamdy, University Urology Unit, Freeman Hospital, Newcastle upon Tyne, NE7 7DN, UK
Received 16 November 1995; revised 12 April 1996; accepted 2 May 1996

prostatectomy specimen. All tumours were clinically and pathologically staged. Grading was performed using the Gleason scoring system. All patients had serum prostate-specific antigen (PSA) measurements before commencement of treatment (Hybritech assay). Patients with advanced and/or metastatic disease were treated by androgen ablation in the form of bilateral orchidectomy or administration of a luteinising hormone-releasing hormone (LHRH) analogue. Men with apparently localised disease were treated by watchful waiting, radical prostatectomy or external beam irradiation. Untreated patients who progressed or those who had radiation therapy and relapsed, received secondary androgen ablation. The follow-up period ranged from 17 to 56 months (median 30 months). Failure to respond to treatment was measured according to the following objective and subjective criteria: (1) less than 50% reduction in serum PSA levels measured at diagnosis; (2) lack of significant alteration in tumour bulk and consistency by digital rectal examination (DRE) and transrectal ultrasonography; and (3) persistent or worsening symptoms directly related to prostate cancer. Progression was defined by one or more of the following observations: (1) a rising serum PSA by at least 50% after initial reduction; (2) new skeletal pain related to metastases as shown by isotope bone scanning; and (3) significant alteration in the primary tumour on DRE.

*Immunohistochemistry and frequency of apoptotic bodies*

Sections (4 μm) were cut from formalin-fixed paraffin-embedded blocks. These were picked up on APES-coated slides, and dried at 60°C for 1 h. Antigen retrieval was carried out in an Energy Beam Sciences H2500 microwave processor. Sections were heated to 95°C in 0.1 M citrate buffer, pH 7.6, and held at this temperature for 20 min before immunostaining. Endogenous peroxidase activity was quenched by incubation in 3% hydrogen peroxidase for 10 min, while other non-specific activity was reduced by incubation in normal goat serum for a further 10 min. A standard streptavidin–biotin complex (DAKO Duet) method was used with sections incubated in optimally diluted primary antisera overnight at 4°C. Both primary antibodies were supplied by DAKO. The anti-p53 DO-7 (Dako, UK Ltd) was diluted to 1:250 in Tris-buffered saline (TBS), and anti-bcl-2 (Dako, UK Ltd) diluted in 1:40 in TBS. About 1000 cells were counted simultaneously by two observers (MCR and IA) to detect p53 nuclear protein accumulation and bcl-2 protein overexpression. The intensity of nuclear p53 protein accumulation was classified according to the percentage of cells with strong nuclear staining: + = 5–25%, + + = 26–75%, + + + = > 75%. Intensity of cytoplasmic staining for bcl-2 in tumour cells was categorised as + = focal areas of strong staining (< 5%); + + = diffuse staining (5–50%); + + + = diffuse staining (> 50%). Positive controls matching the fixation protocol of the test material were used. These were colorectal carcinoma for p53 and tonsil for bcl-2. In addition, basal cells in benign prostatic glands and lymphocytes, which are known to stain positively for bcl-2, were used as internal positive control. Negative controls were performed by omiting the primary antibody in each case. Representative sections from 51 cancer specimens and ten BPH tissue samples were analysed for frequency of apoptotic bodies. For each case, a single representative slide (haematoxylin and eosin stained) was chosen for grading and counting of apoptotic bodies by two observers (MCR and NMW). Apoptotic bodies were defined according to established criteria (Searle *et al*., 1982). Areas of well-preserved tumour corresponding to positive staining for p53 and/or bcl-2 were assessed. An eyepiece graticule was used to define the area selected for counting. All interphase tumour nuclei and apoptotic bodies in the field were counted. The

**Figure 1** (**a**) Photomicrograph of a tissue section showing prostatic adenocarcinoma with strong nuclear staining (+ + +) for p53 (magnification approximately ×125). (**b**) Photomicrograph of a tissue section showing prostatic adenocarcinoma with positive cytoplasmic staining for bcl-2 (magnification approximately ×400).

process was repeated until a total of 2000 interphase nuclei were counted. The number of apoptotic bodies detected was divided by 2 to give an apoptotic index (number of apoptotic bodies per 1000 interphase nuclei). Fisher's exact test and Kendall rank correlation were used for statistical analysis of the results. *P*-values less than 0.05 were considered statistically significant.

### Results

### *Patient outcome*

Forty-five patients (58.4%) had metastatic disease confirmed by isotope bone scanning, and were treated by means of hormone manipulation. Fourteen men had locally advanced disease with no evidence of metastasis and 11 were treated with external beam irradiation. The other three patients received watchful waiting. Eighteen patients had clinically localised disease. Of those, one had a radical prostatectomy (T2aN0M0, Gleason score 5, negative margins and no extracapsular extension), seven received radiotherapy, and the remaining ten were observed.

Six of the 45 men treated by means of hormone manipulation (13.3%) failed to respond to androgen ablation, and died within 6 months from diagnosis. Of the remaining 39 patients, 20 (51.3%) relapsed within 37 months from the start of treatment (median 20 months). Seven of 18 patients treated by means of radiotherapy relapsed within 22 months from treatment (median 13 months), and were treated by means of secondary androgen ablation. Of those, two men failed to respond. Of the 13 patients on watchful waiting, two died of disease progression and four from other unrelated causes. The remaining seven men are alive and well. The patient who had a radical prostatectomy is alive, well and disease free with an undetectable serum PSA 56 months following surgery.

### *Immunostaining and apoptotic index (AI)*

Thirty-seven of the 77 cancers (48%) showed cytoplasmic staining for bcl-2 (Figure 1b) (+, $n=17$; + +, $n=14$; + + +, $n=6$). Twenty-seven of these 37 patients were treated with hormone manipulation and 20 (74%) showed hormone resistance ($P=0.0128$), either initially ($n=5$) or through escape ($n=15$). Twenty-three of 77 men (29.8%) showed strong nuclear staining for p53 (Figure 1a) (+, $n=3$; + +, $n=11$; + + +, $n=9$). Of those, 21 were treated with hormones and 14 (66.6%) were hormone resistant ($P=0.0012$) initially ($n=4$), or showed progression ($n=12$). Seventeen patients showed both bcl-2 overexpression and p53 nuclear accumulation, 16 of whom were hormonally manipulated, with 13 (81.3%) having hormone-resistant disease ($P<0.0001$), at the onset of treatment ($n=4$), or by relapse ($n=9$). Figures 2–4 illustrate the distribution of bcl-2/p53 immunostaining in patients undergoing hormonal therapy and their response to treatment. There were no statistically significant differences between different groups of patients who expressed bcl-2 and/or p53 in terms of serum PSA, Gleason scores, tumour stage and response to radiotherapy (data not shown). In the 51 cancer patients assessed for frequency of apoptotic bodies, AI ranged from 0.5 to 24.5 (mean 2.1) and showed a statistically significant correlation with high tumour grade ($P<0.001$). In men with BPH, AI ranged from 0–0.5 (mean 0.3). There was no significant correlation between AI and immunostaining for either p53 and/or bcl-2 proteins.

### Discussion p53 protein accumulation and bcl-2 overexpression have been investigated independently in a large number of different malignancies. In prostate cancer, p53 nuclear staining appears to be present in approximately 20% of tumours (Navone *et al.*, 1993; Visakorpi *et al.*, 1992; Mellon *et al.*, 1992) with occasional discrepancies, often attributed to differences in antibodies used, the dilutions of these antibodies, fixation and processing of the tissues, stage and grade of the tumours investigated and finally, interpretation of the staining patterns (Fisher *et al.*, 1994). Several reports also confirm that nuclear p53 accumulation correlates with hormone-resistant and aggressive disease (Navone *et al.*, 1993; Hamdy *et al.*, 1993; Aprikian *et al.*, 1994; Myers *et al.*, 1994). Two further developments have heightened the interest in p53 and its relationship with aggressive prostate cancer. Firstly, the regulating effect of wild-type p53 on apoptosis, and secondly, the finding that tumour regression in prostate cancer following hormone manipulation is largely mediated by programmed cell death (Kyprianou *et al.*, 1990). This led

**Figure 2** bcl-2 expression and response to treatment following hormone manipulation ($n=45$).

**Figure 3** p53 nuclear protein accumulation and response to treatment following hormone manipulation ($n=45$).

**Figure 4** Combined bcl-2/p53 staining and response to treatment following hormone manipulation ($n = 45$).

researchers to investigate other possible mechanisms by which apoptosis could be deregulated in prostate cancer, resulting in progression and hormonal escape, which to date, is an incurable condition.

bcl-2 is an oncogene that prolongs cell viability by preventing and overriding programmed cell death mechanisms. bcl-2 overexpression has been demonstrated in hormone-independent prostate cancer in a number of studies. In particular, McDonnell *et al.* (1992) have found a strong correlation between bcl-2 overexpression by immunohistochemistry and progression of prostate cancer from androgen dependence to hormone resistance in humans. A more recent study by Raffo *et al.* (1995) demonstrated that bcl-2 overexpression protected prostate cancer cell lines (LNCaP) against apoptotic stimuli *in vitro*, and enabled the cells to form tumours in castrated male nude mice *in vivo*, supporting the hypothesis that bcl-2 may be an important factor in the development of hormone-resistant disease. In contrast with prostate cancer, several studies of bcl-2 in other malignancies including colorectal, breast and lung cancer have shown a correlation between bcl-2 protein expression and favourable outcome (Öfner *et al.*, 1995; Pezzella *et al.*, 1993; Leek *et al.*, 1994). There is, however, a growing body of evidence linking bcl-2 and p53 interaction with regulation of a common cell death pathway. Observations have been made that bcl-2 is able to block p53-associated apoptosis in transformed cell lines. In high-grade B lymphomas Piris *et al.* (1994) have demonstrated that simultaneous expression of bcl-2 and p53 protein was associated with poorer prognosis than p53 accumulation alone. Marin *et al.* (1994) also found that bcl-2 and p53 may act as potential regulators of a common apoptotic pathway in lymphomagenesis, and demonstrated that overexpressed bcl-2 suppressed wild-type p53-associated apoptosis following $\gamma$-irradiation. Furthermore, Haldar *et al.* (1994) have demonstrated a possible novel mechanism for p53-induced apoptosis through down-regulation of bcl-2. In prostate cancer, the relevance of this phenomenon remains controversial. A recent report by Berges *et al.* (1993) suggests that p53 gene expression is not required to mediate programmed cell death in androgen-deprived prostatic glandular epithelial cells, and a further study showed that in rat androgen-sensitive prostatic adenocarcinoma, the biochemical cascade leading to apoptosis was not activated by androgen withdrawal, as in the ventral prostate (Bränström *et al.*, 1994).

This information has prompted us to investigate p53 protein accumulation and bcl-2 overexpression simultaneously in a series of patients with different stages of untreated prostate cancer, and to correlate immunohistochemical findings with frequency of apoptotic bodies and clinical data collected from each patient, including response to treatment. Our results show that approximately 50% of primary prostatic tumours overexpress bcl-2, in accordance with previous studies, but a higher percentage of tumours (30%) were found to stain positively for nuclear p53 compared with the majority of other published series including a previous study from our own institutions (Mellon *et al.*, 1993; Hamdy *et al.*, 1993). This may reflect differences in technique and the recent adoption of antigen retrieval methods in our pathology departments. It is also important to note that the antibody used has the ability to detect both wild and mutant forms of p53, which is a known limitation of immunohistochemical studies (Wynford-Thomas, 1992; Hall and Lane, 1994). We have not attempted to use other antibodies to detect p53 protein, in view of recent evidence suggesting that DO7 is the most sensitive and specific currently available antibody when correlated with p53 mutations (Baas *et al.*, 1994). In accordance with previous work, frequency of apoptotic bodies correlated significantly with high tumour grade (Aihara *et al.*, 1994), but did not appear to be altered by the presence or absence of p53 and/or bcl-2 immunostaining. However, the tissue samples analysed in our study were obtained from newly diagnosed and untreated patients, which may explain the lack of correlation with bcl-2 overexpression and p53 protein accumulation. We have not been able to collect tissue from hormonally treated men for ethical reasons. It is this specific group of men, in particular those with clinical signs of hormonal resistance or escape, who may show alterations of apoptotic index in relation to their bcl-2/p53 immunohistochemical status. This putative correlation has yet to be proven. When analysed individually, both p53 and bcl-2 overexpression appear to correlate independently with hormone-resistant disease. When combined, this effect appears to be synergistic, illustrated by the fact that over 80% of patients showed hormonal resistance and/or escape with high statistical significance.

Fifty years after the work of Charles Huggins (Huggins and Hodges, 1941), the mechanisms of hormone resistance in prostate cancer are still poorly understood. Our study has attempted to shed some light on this phenomenon. Despite the many unexplained complexities relating immunostaining with genetic alterations, our results suggest that the combined detection of p53 protein accumulation and bcl-2 overexpression may be useful in the prediction of hormone-resistant disease in prostate cancer. Ongoing studies at the molecular level may clarify some of the mechanisms controlling hormone dependence of this common, yet unpredictable, malignancy.

**Acknowledgements**
Dr JA Royds is supported by the Yorkshire Cancer Research Campaign.

### References

AIHARA M, TRUONG LD, DUNN JK, WHEELER TM, SCARDINO PT AND THOMPSON TC. (1994). Frequency of apoptotic bodies positively correlates with Gleason grade in prostate cancer. *Hum. Pathol.*, **25**, 797–801.

APRIKIAN AG, SARKIS AS, FAIR WR, ZHANG Z-F, FUKS Z AND CORDON-CARDO C. (1994). Immunohistochemical determination of p53 protein nuclear accumulation in prostatic adenocarcinoma. *J. Urol.*, **151**, 1276–1280.

BAAS IO, MULDER J-WR, OFFERHAUS JA, VOGELSTEIN B AND HAMILTON SR. (1994). An evaluation of six antibodies for immunohistochemistry of mutant p53 gene product in archival colorectal neoplasms. *J. Pathol.*, **172**, 5–12.

BERGES RR, FURUYA Y, REMINGTON L, ENGLISH HF, JACKS T AND ISAACS JT. (1993). Cell proliferation, DNA repair, and p53 function are not required for programmed cell death of prostatic glandular cells induced by androgen ablation. *Proc. Natl Acad. Sci. USA*, **90**, 8910–8914.

BISHOP JM. (1991). Molecular themes in carcinogenesis. *Cell*, **64**, 235–248.

BISSONNETTE RP, ECHEVERRI F, MAHBOUBI A AND GREEN DR. (1992). Apoptotic cell death induced by c-*myc* is inhibited by *bcl*-2. *Nature*, **359**, 552–554.

BRÄNDSTRÖM A, WESTIN P, BERGH A, CAJANDER S AND DAMBER J-E. (1994). Castration induces apoptosis in the ventral prostate but not in an androgen-sensitive prostatic adenoscarcinoma in the rat. *Cancer Res.*, **54**, 3594–3601.

COLOMBEL M, OLSSON CA, NG P-Y AND BUTTYAN R. (1992). Hormone-regulated apoptosis results from reentry of differentiated prostate cells onto a defective cell cycle. *Cancer Res.*, **52**, 4313–4319.

COLOMBEL M, SYMMANS F, GIL S, O'TOOLE KM, CHOPIN D, BENSON M, OLSSON C, KORSMEYER S AND BUTTYAN R. (1993). Detection of apoptosis suppressing oncoprotein bcl-2 in hormone-refractory human prostate cancers. *Am. J. Pathol.*, **143**, 390–400.

FISHER CJ, GILLETT CE, VOJTESEK DM, BARNES DM AND MILLIS RR. (1994). Problems with p53 immunohistochemical staining: the effect of fixation and variation in the methods of evaluation. *Br. J. Cancer*, **69**, 20–31.

HALDAR S, NEGRINI M, MONNE M, SABBIONI S AND CROCE CM. (1994). Down-regulation of *bcl*-2 by *p53* in breast cancer cells. *Cancer Res.*, **54**, 2095–2097.

HALL PA AND LANE DP. (1994). p53 in tumour pathology: can we trust immunohistochemistry? – Revisited! *J. Pathol.*, **172**, 1–4.

HAMDY FC, THURRELL W, LAWRY J, ANDERSON JB, PARSONS MA, REES RC AND ROYDS JA. (1993). p53 mutant expression correlated with hormone sensitivity and prognosis in human prostatic adenocarcinoma. *J. Urol.*, **149**, 377A.

HOCKENBURY D, NUÑEZ G, MILLIMAN C, SCHREIBER RD AND KORSMEYER SJ. (1990). Bcl-2 is an inner mitochondrial membrane protein that blocks programmed cell death. *Nature*, **348**, 334–336.

HUGGINS C AND HODGES CV. (1941). Studies on prostate cancer: The effect of castration, of oestrogen and of androgen injection on serum phosphatase in metastatic carcinoma of the prostate. *Cancer Res.*, **1**, 293–297.

KERR JFR, WINTERFORD CM AND HARMON BV. (1994). Apoptosis. Its significance in cancer and cancer therapy. *Cancer*, **73**, 2013–2026.

KORSMEYER SJ. (1992). *Bcl*-2 initiates a new category of oncogenes: regulators of cell death. *Blood*, **80**, 879–886.

KYPRIANOU N, ENGLISH HF AND ISAACS JT. (1990). Programmed cell death during regression of PC-82 human prostate cancer following androgen ablation. *Cancer Res.*, **50**, 3748–3753.

LANE DP. (1992). p53, guardian of the genome. *Nature*, **358**, 15–16.

LEEK RD, KAKLAMANIS L, PEZZELLA F, GATTER KC AND HARRIS AL. (1994). bcl-2 in normal human breast and carcinoma, associated with oestrogen receptor-positive, epidermal growth factor receptor-negative tumours and *in situ* cancer. *Br. J. Cancer*, **69**, 135–139.

MCDONNELL TJ, TRONCOSO P, BRISBAY SM, LOGOTHETIS C, CHUNG LWK, HSIER J-T, TU S-M AND CAMPBELL ML. (1992). Expression of the protooncogene bcl-2 in prostate and its association with emergence of androgen-independent prostate cancer. *Cancer Res.*, **52**, 6940–6944.

MARIN MC, HSU B, MEYN RE, DONEHOWER LA, EL-NAGGAR AK AND MCDONNELL TJ. (1994). Evidence that p53 and bcl-2 are regulators of a common cell death pathway important for *in vivo* lymphomagenesis. *Oncogene*, **9**, 3107–3112.

MARSHALL C. (1991). Tumor suppressor genes. *Cell*, **64**, 313–326.

MELLON K, THOMPSON S, CHARLTON RG, MARSH C, ROBINSON M, LANE DP, HARRIS AL, HORNE CHW AND NEAL DE. (1992). p53, c-erb and the epidermal growth factor receptor in the benign and malignant prostate. *J. Urol.*, **147**, 496–499.

MYERS RB, OELSCHLAGER D, SRIVASTAVA S AND GRIZZLE WE. (1994). Accumulation of the p53 protein occurs more frequently in metastatic than localized prostatic adenocarcinomas. *The Prostate*, **25**, 243–248.

NAVONE N, TRONCOSO P, PISTERS LL, GOODROW TL, PALMER JL, NICHOLS WW, VON ESCHENBACH AC AND CONTI CJ. (1993). p53 protein accumulation and gene mutation in the progression of human prostate carcinoma. *J. Natl Cancer Inst.*, **85**, 1657–1699.

OFFICE OF POPULATION CENSUSES AND SURVEYS. (1993). *Cancer Statistics. Registrations 1987. England and Wales*. Series MB1 No.20. HMSO: London.

ÖFNER D, RIEHEMANN K, MAIER H, REIDMANN B, NEHODA H, TÖTSCH M, BÖCKER W, JASANI B AND SCHMID KW. (1995). Immunohistochemically detectable bcl-2 expression in colorectal carcinoma: correlation with tumour stage and patient survival. *Br. J. Cancer*, **72**, 981–985.

PEZZELLA F, TURLEY H, KUZU I, TUNGEKAR MF, DUNNILL MS, PERCE CB, HARRIS A, GATTER K AND MASON D. (1993). Bcl-2 protein in non-small-cell lung carcinoma. *N. Engl. J. Med.*, **329**, 690–694.

PIRIS MA, PEZZELLA F, MARTINEZ-MONTERO JC, ORRADRE J., VILLUENDAS R, SANCHEZ-BEATO M, CUEN AR, CRUZ MA, MARTINEZ B, GARRIDO MC, GATTER K, AIELLO A, DELIA D, GIARDINI R AND RILKE F. (1994). p53 and bcl-2 expression in high grade B-cell lymphomas: correlation with survival time. *Br. J. Cancer*, **69**, 337–341.

RAFFO AJ, PERLMAN H, CHEN M-W, DAY ML, STREITMAN JS AND BUTTYAN R. (1995). Overexpression of bcl-2 protects prostate cancer cells from apoptosis *in vitro* and confers resistance to androgen depletion *in vivo*. *Cancer Res.*, **55**, 4438–4445.

SEARLE J, KERR JFR AND BISHOP CJ. (1982). Necrosis and apoptosis: distinct mode of cell death with fundamentally different significance. *Pathol. Ann.*, **17**, 229–259.

SHAW P, BOVEY R, TARDY S, SAHLI R, SORDAT B AND COSTA J. (1992). Induction of apoptosis by wild-type p53 in a human colon tumor-derived cell line. *Proc. Natl Acad. Sci. USA*, **89**, 4495–4499.

VISAKORPI T, KALLIONIEMI O-P, HEIKKINEN A, KOIVULA T AND ISOLA J. (1992). Small subgroup of aggressive highly proliferative prostatic carcinomas defined by p53 accumultaion. *J. Natl Cancer Inst.*, **84**, 883.

WYNFORD-THOMAS D. (1992). p53 in tumour pathology: can we trust immunohistochemistry? *J. Pathol.*, **166**, 329–330.


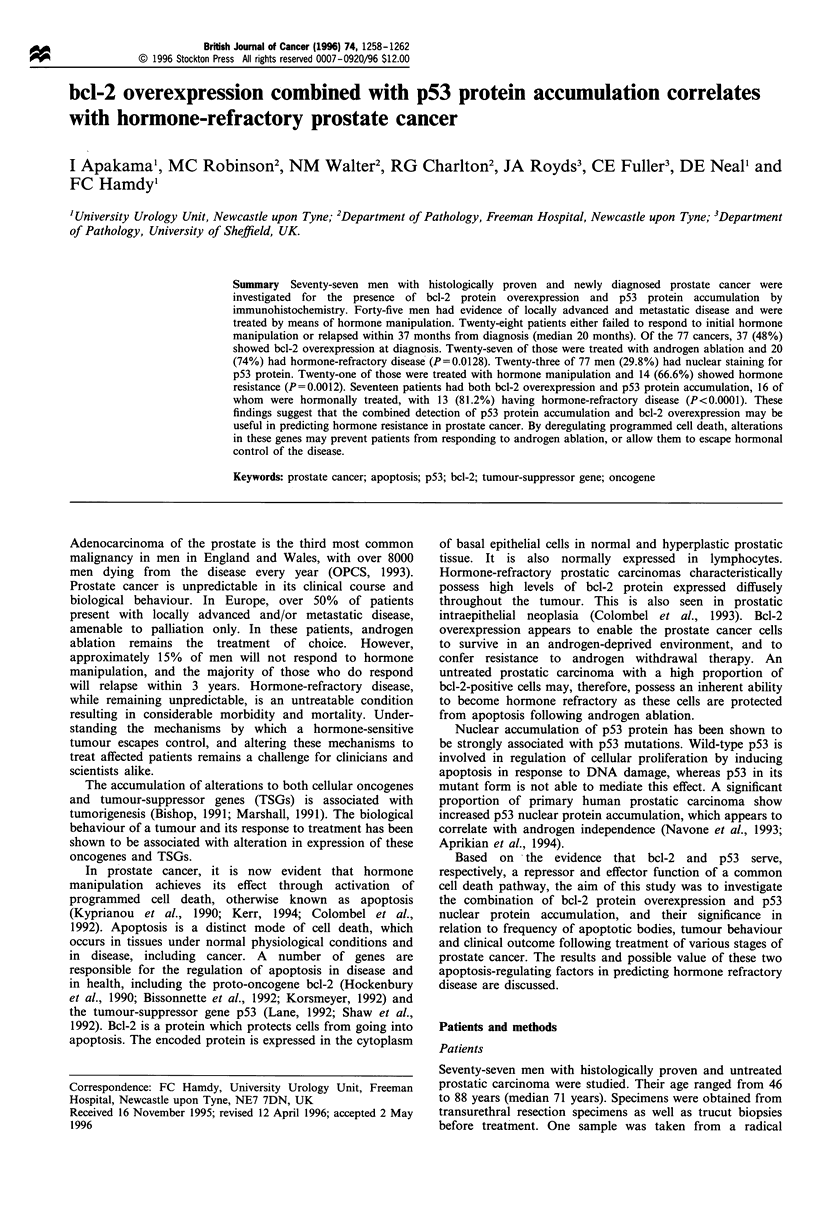

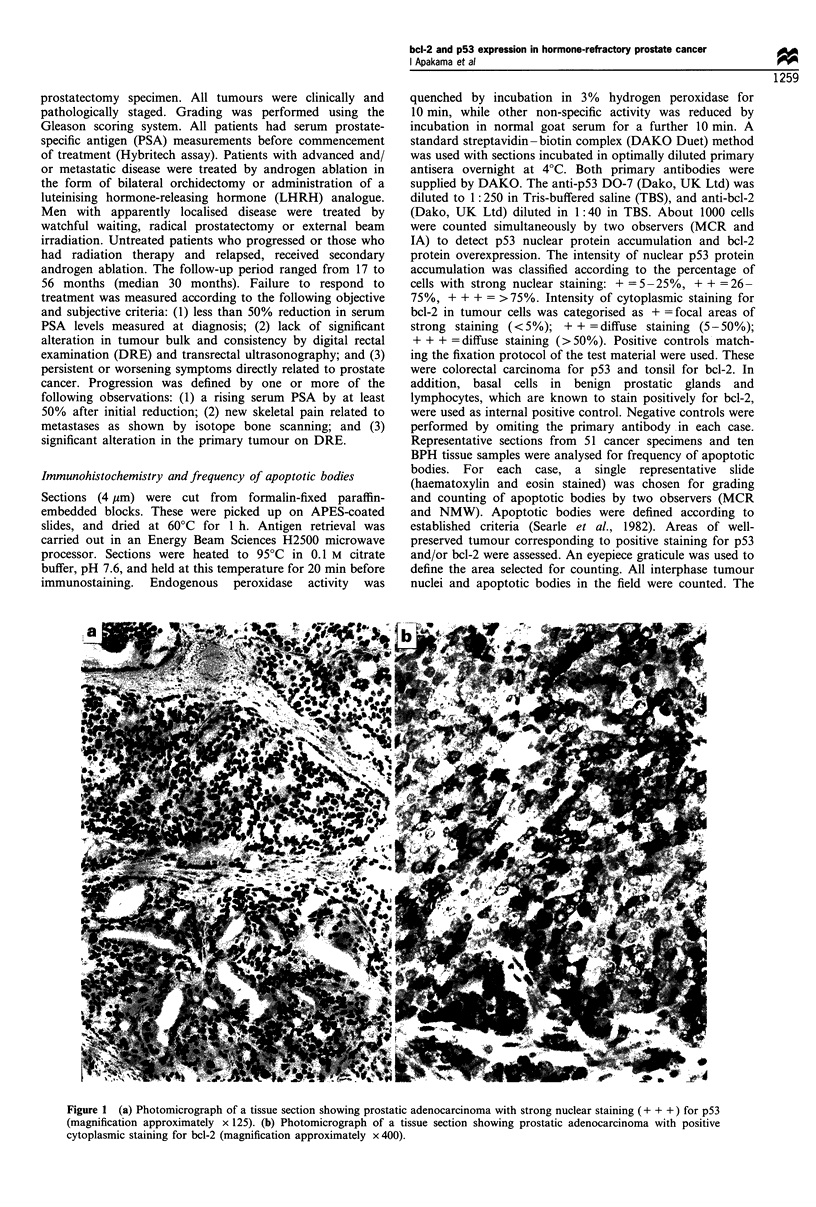

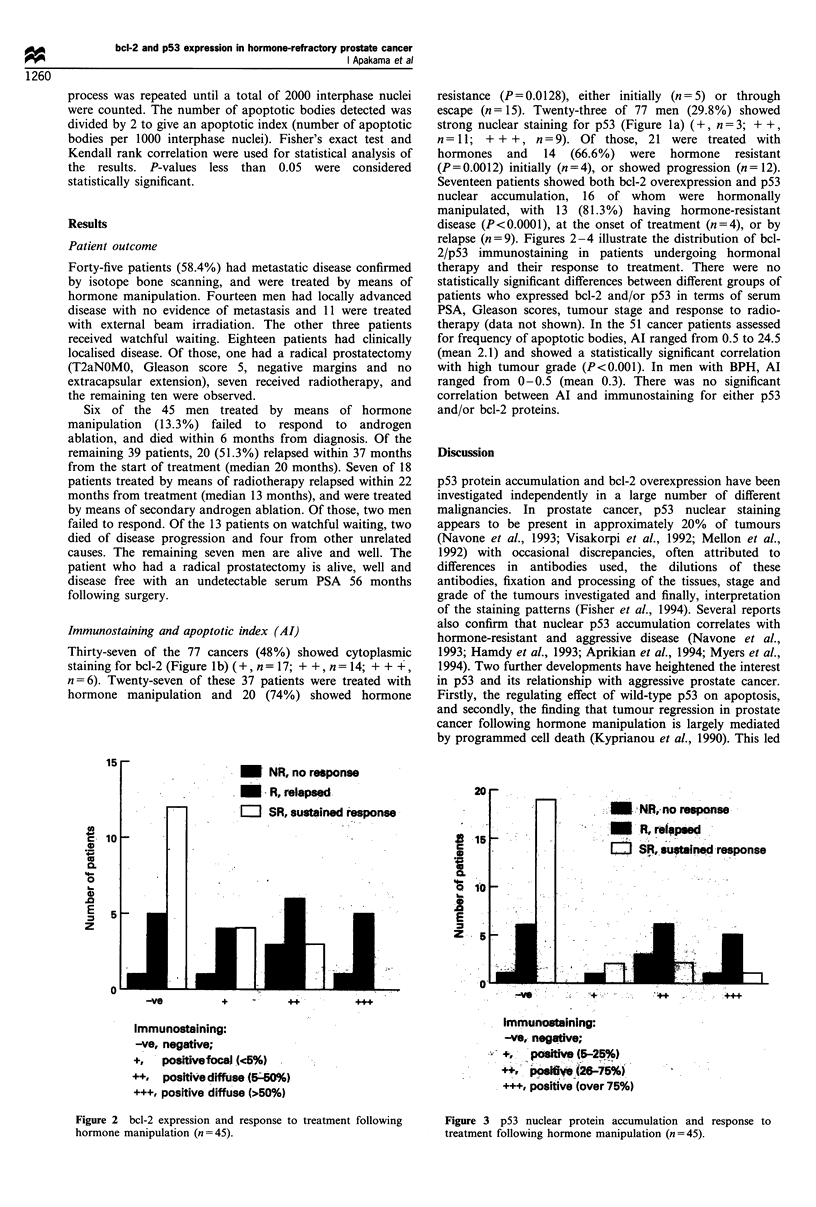

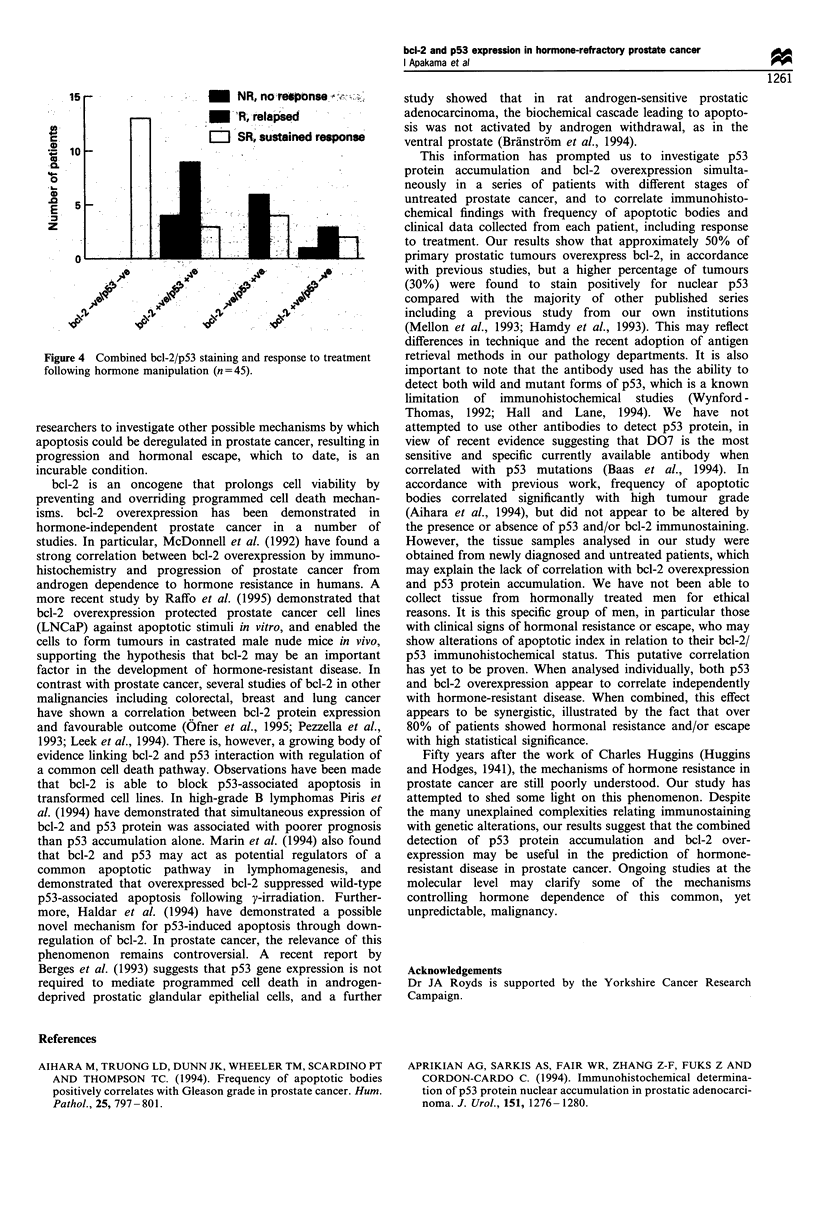

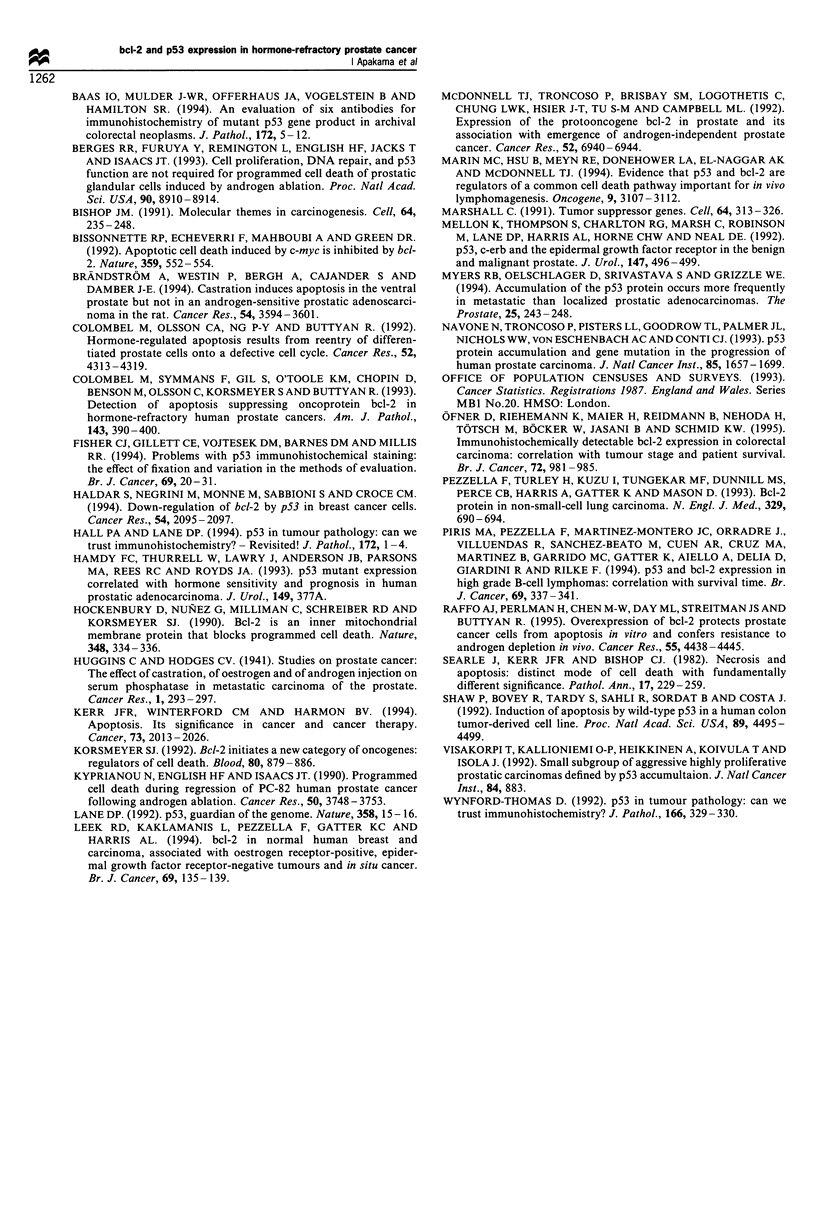


## References

[OCR_00514] Aihara M., Truong L. D., Dunn J. K., Wheeler T. M., Scardino P. T., Thompson T. C. (1994). Frequency of apoptotic bodies positively correlates with Gleason grade in prostate cancer.. Hum Pathol.

[OCR_00521] Aprikian A. G., Sarkis A. S., Fair W. R., Zhang Z. F., Fuks Z., Cordon-Cardo C. (1994). Immunohistochemical determination of p53 protein nuclear accumulation in prostatic adenocarcinoma.. J Urol.

[OCR_00531] Baas I. O., Mulder J. W., Offerhaus G. J., Vogelstein B., Hamilton S. R. (1994). An evaluation of six antibodies for immunohistochemistry of mutant p53 gene product in archival colorectal neoplasms.. J Pathol.

[OCR_00534] Berges R. R., Furuya Y., Remington L., English H. F., Jacks T., Isaacs J. T. (1993). Cell proliferation, DNA repair, and p53 function are not required for programmed death of prostatic glandular cells induced by androgen ablation.. Proc Natl Acad Sci U S A.

[OCR_00543] Bishop J. M. (1991). Molecular themes in oncogenesis.. Cell.

[OCR_00547] Bissonnette R. P., Echeverri F., Mahboubi A., Green D. R. (1992). Apoptotic cell death induced by c-myc is inhibited by bcl-2.. Nature.

[OCR_00553] Brändström A., Westin P., Bergh A., Cajander S., Damber J. E. (1994). Castration induces apoptosis in the ventral prostate but not in an androgen-sensitive prostatic adenocarcinoma in the rat.. Cancer Res.

[OCR_00558] Colombel M., Olsson C. A., Ng P. Y., Buttyan R. (1992). Hormone-regulated apoptosis results from reentry of differentiated prostate cells onto a defective cell cycle.. Cancer Res.

[OCR_00564] Colombel M., Symmans F., Gil S., O'Toole K. M., Chopin D., Benson M., Olsson C. A., Korsmeyer S., Buttyan R. (1993). Detection of the apoptosis-suppressing oncoprotein bc1-2 in hormone-refractory human prostate cancers.. Am J Pathol.

[OCR_00577] Haldar S., Negrini M., Monne M., Sabbioni S., Croce C. M. (1994). Down-regulation of bcl-2 by p53 in breast cancer cells.. Cancer Res.

[OCR_00592] Hockenbery D., Nuñez G., Milliman C., Schreiber R. D., Korsmeyer S. J. (1990). Bcl-2 is an inner mitochondrial membrane protein that blocks programmed cell death.. Nature.

[OCR_00602] Kerr J. F., Winterford C. M., Harmon B. V. (1994). Apoptosis. Its significance in cancer and cancer therapy.. Cancer.

[OCR_00607] Korsmeyer S. J. (1992). Bcl-2 initiates a new category of oncogenes: regulators of cell death.. Blood.

[OCR_00613] Kyprianou N., English H. F., Isaacs J. T. (1990). Programmed cell death during regression of PC-82 human prostate cancer following androgen ablation.. Cancer Res.

[OCR_00617] Leek R. D., Kaklamanis L., Pezzella F., Gatter K. C., Harris A. L. (1994). bcl-2 in normal human breast and carcinoma, association with oestrogen receptor-positive, epidermal growth factor receptor-negative tumours and in situ cancer.. Br J Cancer.

[OCR_00631] Marin M. C., Hsu B., Meyn R. E., Donehower L. A., el-Naggar A. K., McDonnell T. J. (1994). Evidence that p53 and bcl-2 are regulators of a common cell death pathway important for in vivo lymphomagenesis.. Oncogene.

[OCR_00637] Marshall C. J. (1991). Tumor suppressor genes.. Cell.

[OCR_00624] McDonnell T. J., Troncoso P., Brisbay S. M., Logothetis C., Chung L. W., Hsieh J. T., Tu S. M., Campbell M. L. (1992). Expression of the protooncogene bcl-2 in the prostate and its association with emergence of androgen-independent prostate cancer.. Cancer Res.

[OCR_00641] Mellon K., Thompson S., Charlton R. G., Marsh C., Robinson M., Lane D. P., Harris A. L., Horne C. H., Neal D. E. (1992). p53, c-erbB-2 and the epidermal growth factor receptor in the benign and malignant prostate.. J Urol.

[OCR_00644] Myers R. B., Oelschlager D., Srivastava S., Grizzle W. E. (1994). Accumulation of the p53 protein occurs more frequently in metastatic than in localized prostatic adenocarcinomas.. Prostate.

[OCR_00653] Navone N. M., Troncoso P., Pisters L. L., Goodrow T. L., Palmer J. L., Nichols W. W., von Eschenbach A. C., Conti C. J. (1993). p53 protein accumulation and gene mutation in the progression of human prostate carcinoma.. J Natl Cancer Inst.

[OCR_00663] Ofner D., Riehemann K., Maier H., Riedmann B., Nehoda H., Tötsch M., Böcker W., Jasani B., Schmid K. W. (1995). Immunohistochemically detectable bcl-2 expression in colorectal carcinoma: correlation with tumour stage and patient survival.. Br J Cancer.

[OCR_00667] Pezzella F., Turley H., Kuzu I., Tungekar M. F., Dunnill M. S., Pierce C. B., Harris A., Gatter K. C., Mason D. Y. (1993). bcl-2 protein in non-small-cell lung carcinoma.. N Engl J Med.

[OCR_00673] Piris M. A., Pezzella F., Martinez-Montero J. C., Orradre J. L., Villuendas R., Sanchez-Beato M., Cuena R., Cruz M. A., Martinez B., Pezella F [corrected to Pezzella F. ]. (1994). p53 and bcl-2 expression in high-grade B-cell lymphomas: correlation with survival time.. Br J Cancer.

[OCR_00684] Raffo A. J., Perlman H., Chen M. W., Day M. L., Streitman J. S., Buttyan R. (1995). Overexpression of bcl-2 protects prostate cancer cells from apoptosis in vitro and confers resistance to androgen depletion in vivo.. Cancer Res.

[OCR_00689] Searle J., Kerr J. F., Bishop C. J. (1982). Necrosis and apoptosis: distinct modes of cell death with fundamentally different significance.. Pathol Annu.

[OCR_00692] Shaw P., Bovey R., Tardy S., Sahli R., Sordat B., Costa J. (1992). Induction of apoptosis by wild-type p53 in a human colon tumor-derived cell line.. Proc Natl Acad Sci U S A.

[OCR_00698] Visakorpi T., Kallioniemi O. P., Heikkinen A., Koivula T., Isola J. (1992). Small subgroup of aggressive, highly proliferative prostatic carcinomas defined by p53 accumulation.. J Natl Cancer Inst.

[OCR_00704] Wynford-Thomas D. (1992). P53 in tumour pathology: can we trust immunocytochemistry?. J Pathol.

